# Nature’s therapeutic power: a study on the psychophysiological effects of touching ornamental grass in Chinese women

**DOI:** 10.1186/s41043-024-00514-6

**Published:** 2024-02-03

**Authors:** Ahmad Hassan, Zhang Deshun

**Affiliations:** College of Architecture and Urban Planning, Tongji University, 1239 Siping Rd, Shanghai, People’s Republic of China

**Keywords:** Real grass, Artificial turf, Stress reduction, Electroencephalography, Urbanization

## Abstract

The health of city residents is at risk due to the high rate of urbanization and the extensive use of electronics. In the context of urbanization, individuals have become increasingly disconnected from nature, resulting in elevated stress levels among adults. The goal of this study was to investigate the physical and psychological benefits of spending time in nature. The benefits of touching real grass and artificial turf (the control activity) outdoors with the palm of the hand for five minutes were measured. Blood pressure and electroencephalography (EEG) as well as State-trait Anxiety Inventory (STAI) scores, and the semantic differential scale (SDM) were used to investigate psychophysiological responses. Touching real grass was associated with significant changes in brainwave rhythms and a reduction in both systolic and diastolic blood pressure compared to touching artificial turf. In addition, SDM scores revealed that touching real grass increased relaxation, comfort, and a sense of naturalness while decreasing anxiety levels. Compared to the control group, the experimental group had higher mean scores in both meditation and attentiveness. Our findings indicate that contact with real grass may reduce physiological and psychological stress in adults.

## Introduction

In the twenty-first century, global health is being affected by urbanization. Over 50% of the world's population lives in cities, and this number is increasing [1, 2]. Modern individuals psychologically suffer from urban life [3]. Urban inhabitants composed 30% of the world population in 1950, while 54% of the globe lived in cities in 2014, and this increasing trend is expected to continue. It is projected that cities will host 66% of the world population by 2050 [4]. Lederbogen et al. contrasted urban and rural amygdala stress; specifically, they examined the differential amygdala activation that occurs during social stress. Urbanites reportedly exhibit greater amygdala activation than ruralites [3]. It has been found that urbanites may overreact to stress [5], and their level of stress is increased by tech stress from cellphones, computers, and social media [6]. Jiang et al. reported that green settings greatly reduce the stress-enhancing effects of electronic devices [7].

Stress, pollution, violence, and social isolation are detrimental to the mental health of urban residents. The WHO predicts that by 2030, 15% of DALYs will be caused by these variables [8]. Many productive young individuals suffer from mental health issues, the most common of which are depression and anxiety. Hunt et al. [9] and Peen et al. [10] reported 21% greater anxiety and a 39% greater incidence of mood disorders in urbanites than in ruralites. However, a considerable treatment gap exists due to the worldwide mental disorder load not having been properly addressed by health services [11].

The concept of connecting urbanites to nature for reducing mental health difficulties is becoming more popular. Regular exposure to nature is essential for maintaining favorable attitudes toward nature [12]. Numerous studies have shown that early exposure to nature increases the love of nature [13]. A US study by Bixler et al. [14] indicated that people who prefer pristine natural places have better cognitive experiences in such settings. According to Hinds and Sparks [15], in the UK, rural children are more nature friendly than urban children are. The above research indicates that early childhood experiences with nature shape our relationship with nature. Cheng and Monroe [13] and Zhang et al. [16] found that direct and indirect contact with nature improve the attitudes and feelings for nature.

Plant-based activities are also known to provide benefits. Horticultural activities improve peer connections, social skills, self-efficacy, emotional intelligence, and depression risk in children [17]. Studies have indicated that horticultural hobbies can reduce depression and anxiety, boost self-esteem, and boost BDNF in older people, which in turn boosts cognition [18]. Horticultural activities improve both upper and lower limb functions and muscle activation [19]. Many studies have examined the effect of green vegetation on physiological and psychological responses. In one study, the effects of white walls and plants were compared. The findings show that the presence of plants increases alpha wave activity, blood pressure, and heart rate [20]. Another study revealed that the presence of plants reduces prefrontal lobe oxyhemoglobin levels. The study participants also reported experiencing physiological relaxation [21]. Plants are known to be comforting, and exposure to nature boosts creativity and focus [22]. Research has shown that indoor natural environments reduce anxiety and tension, increase alpha and beta activity, and lower blood pressure [23].

Skin contact with different surfaces can evoke sensory and emotional responses [24]. The sensory components include touch discrimination among pressure, vibration, sliding, and texture. The emotional characteristics of tactile stimuli include pleasant and negative emotions [25]. Touch evaluation frequently emphasizes surface characteristics and subjective experiences. Touch influences preferences, health, and product satisfaction [26]. Many studies have been conducted to evaluate how material surface sensory characteristics affect emotional touch assessment [24].

However, tactile stimuli have rarely been examined. Morikawa et al. [27] found that Japanese cypress wood and artificial plates had different impacts on blood pressure. Sakuragawa et al. [28] studied how tactile stimuli (chilly, room temperature, and warm materials) affected human physiological responses. The researchers conducted a series of observations that yielded the following findings. First, the act of touching an aluminum plate resulted in a rise in blood pressure. However, this increase was mitigated when the aluminum plate was warm. Second, touching an acrylic plastic plate led to an elevation in blood pressure, and this effect was more pronounced when the acrylic plate was cold. Third, touching surfaces composed of Japanese cypress, Japanese cedar, or oak did not elicit any discernible changes in blood pressure, even when the oak surface was cold. Their study aimed to examine the physiological impacts of tactile stimuli through contact with wood on the human body. However, tactile stimuli research is scarce. The activity of planting has been shown to improve cognition more than the control condition [29]. Studies have shown that planting reduces psychophysiological stress compared to cerebral tasks [30]. The precise physiological and psychological responses to tactile stimuli originating from natural sources remain unknown despite empirical studies that have shown a favorable influence of nature on human well-being. Prior research has utilized methods such as measurements of heart rate, heart rate variability, and the Profile of Mood States (POMS) scores [31]. However, few electroencephalography (EEG) studies have examined the stress-reducing effects of tactile stimuli using grass. EEG is an accurate, noninvasive, non-fatiguing, and inexpensive method. EEG can also be used to study the impacts of auditory, gustatory, visual, and olfactory stimuli [32], and EEG devices have also been employed in studies of the brain–computer interface [32] and stress, anxiety, and other psychiatric diagnoses [33]. EEG measurements include activity in delta, beta, theta, alpha, and gamma waves. Beta waves replace alpha waves when mental stress or workload increases [34]. Work stress decreases alpha waves and increases theta waves, according to several studies [35]. Thus, EEG activity varies depending on local conditions, which enhances its precision in evaluating neurophysiological behaviors in human subjects [36]. In a study conducted in 1996, Brookings et al. [37] employed EEG to investigate variations in cognitive workload and stress levels that may not be discernible by alternative measurement methods.

A vast majority of urban residents highly value urban parks and woods that offer extensive landscapes including grass, trees, and open spaces [38]. The absence of green grass areas in parks, alongside boulevards, and surrounding residential areas, educational institutions, commercial establishments, and office spaces can substantially contribute to the overall gloominess of urban environments. Potential consequences include decreased productivity, heightened vulnerability to anxiety, and the development of mental disorders. Ulrich [39] illustrated this phenomenon, demonstrating that exposure to natural outdoor scenery facilitated recuperation among people receiving medical care in a hospital setting [40]. In their seminal work, Kaplan and Kaplan [41] examined the importance of natural elements, such as parks, wooded areas, and expansive landscapes, in enhancing the overall well-being and quality of life of urban residents [42]. They examined the utilization of natural amenities for both recreational purposes and aesthetic enjoyment, including the appreciation of natural beauty. Additionally, the participants in the study indicated a heightened level of contentment with their residential area and an overall improvement in well-being when they were near a natural landscape. Ultimately, greater personal contentment was observed when people actively engaged in gardening endeavors, such as the maintenance and nurturing of the surrounding environment [43]. Recreation and leisure activities on grass areas are highly important in modern society, particularly in highly populated urban regions, in terms of both the pleasure they provide and the benefits they offer for physical and mental well-being [44].

Ornamental grasses serve a crucial function in landscape design, augmenting aesthetic allure and contributing to cultural and historical relevance [45]. In addition, they make a substantial contribution to the diversity of landscapes and play a crucial role in preserving ecological equilibrium. The integration of ornamental grasses in lawns augments the aesthetic allure of recreational and residential spaces within urban environments [46]. The primary objective of this study was to evaluate the psychophysiological effects of tactile stimulation through the act of touching real grass (using the palm) and artificial turf (the control) in the Chinese population by evaluating numerous factors, including blood pressure, EEG activity, and emotional responses.

## Methods

### Participants

The present study recruited 54 female Chinese university students. The participants had an average age of 22.51 years with a standard deviation of 3.61 years. The participants' average weight was 57.9 kg with a standard deviation of 11.0 kg. Additionally, their average height was 164.75 cm with a standard deviation of 6.78 cm. None of the individuals reported a prior medical or psychiatric condition. Individuals with any physical diseases or psychiatric disorders were excluded from the study. In addition, we implemented measures to account for the influence of tobacco and alcohol consumption. To minimize the risk of influencing the outcomes, the purpose of the study was not disclosed to the participants. The current investigation was conducted within the university's laboratory facility number 5, which is located on campus. A single room with white walls and two windows on the southern side was chosen. To minimize external disturbances, the room was kept at a constant temperature and was quiet to ensure an environment conducive to the students' concentration and focus. To minimize potential disruptions during the experiment, all measurement devices were positioned behind the participants. The experiment was conducted during daylight hours, and the temperature, humidity, and light intensity conditions were maintained at 26 °C, 57% relative humidity, and 500 lx, respectively. Written informed consent was obtained from the participants, and the present investigation was approved by the Ethics Committee of the College of Architecture and Urban Planning at Tongji University in China. In this study, consent was obtained from participants to include their photographs in the manuscript.

### Protocol

The research utilized a within-subject design to examine the physiological reactions of the participants in relation to two tasks. The participants were divided into two groups, Group A and Group B. On the first day, group A (*N* = 27) engaged in a task involving the tactile exploration of real grass using the palm of their hand for a duration of 5 min. While group B (*N* = 27) performed a control task (the same movements with artificial turf instead of real grass) for the same duration. On the next day, each group switched tasks. Both experimental activities were conducted while the participants had their eyes closed and maintained the same seated position.

### Materials

**Fig. 1:**
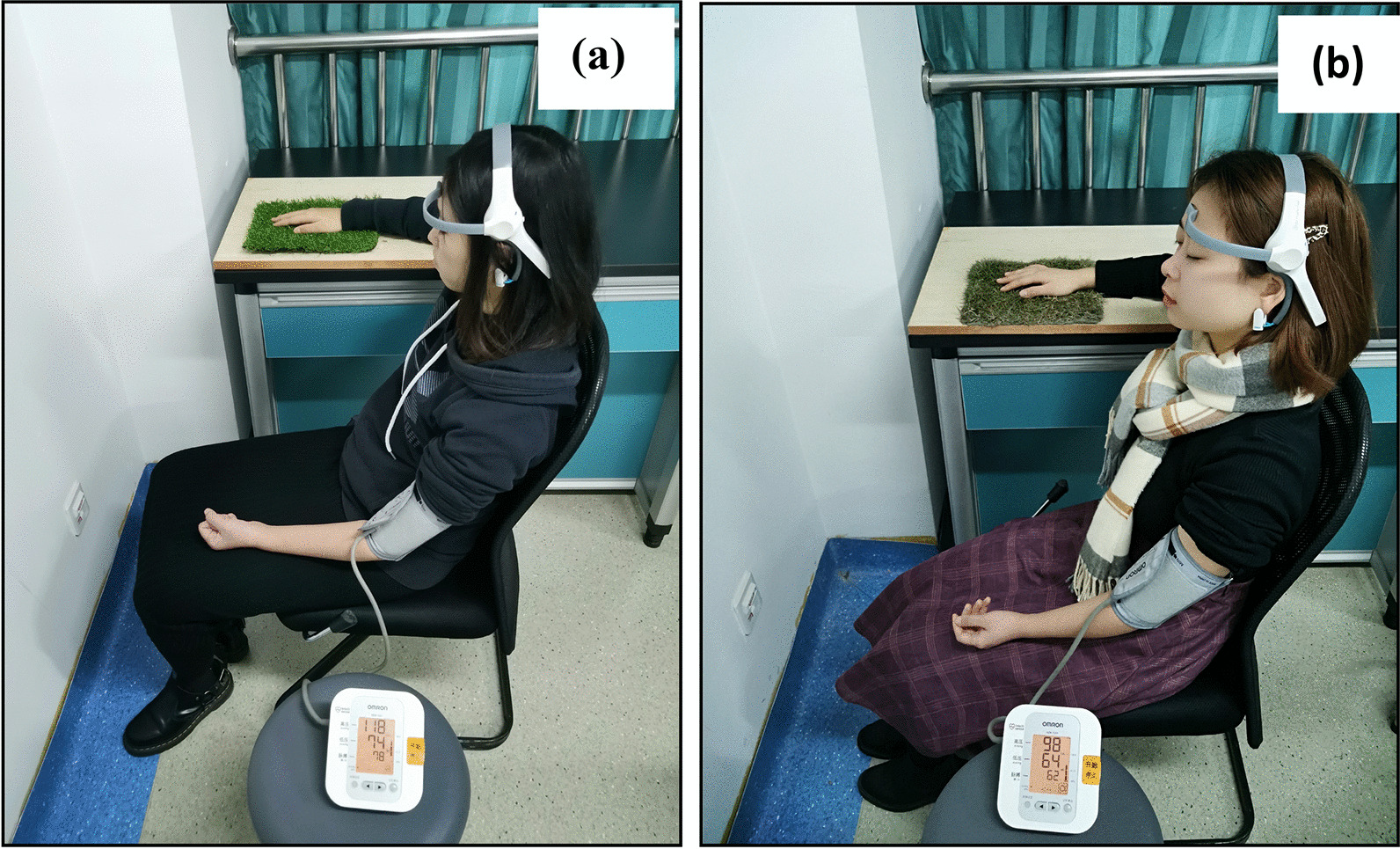
Tactile stimulation (with the palm of the hand) **a** artificial grass (control); **b** real grass

For the purpose of tactile stimulation, a popular Chinese ornamental grass cultivated in a university greenhouse was used. As shown in Fig. 1, the experimental condition was compared with a control condition involving artificial plastic grass of a similar weight and size but purchased from outside the university. The size of the natural and synthetic grasses was approximately 1 × 1.5 ft, and the height of the natural and synthetic grasses was 1.6 inches.

### Measurements

Before the tactile stimuli were presented, subjects completed questionnaires that collected information on their age, weight, and height, the State-Trait Anxiety Inventory (STAI), and the semantic differential scale (SDS). Before the experiment, all participants closed their eyes and touched a white paper sheet on the floor with their palms for 10 s. Each participant was given a physiological measuring device, and after 5 min of rest, their initial blood pressure reading was taken on their right arm in the seated posture. Subsequently, the participants were directed to shut their eyes for a duration of 10 s and proceed to touch either real or synthetic grass material using their palms for a period of 5 min. The EEG data were obtained utilizing the MindWave-EEG headset (MW001) produced by NeuroSky Beijing Oriental Creation Technology Co., Ltd. in Beijing, China. The EEG headset had the capability to record brainwave activity originating from the frontal lobe region, namely, at FP1, a location positioned above the eye on the human forehead [47]. The system comprised four distinct components, specifically, a Bluetooth device, an ear clip, a sensor arm housing the EEG electrode, and a headband. The device was outfitted with a pair of dry sensors that were capable of detecting and filtering EEG data. The sensor tip had the capability to detect and analyze electrical signals captured from the frontal region of the human head. Additionally, this sensor could detect ambient noise originating from human musculature, lighting fixtures, electrical outlets, and various other electrical apparatuses. The ear clip served a dual purpose, functioning as a grounding mechanism and a reference point, enabling the chip (ThinkGear) to effectively eliminate any interference caused by electrical noise [48]. The power spectrum captured the unprocessed signal, encompassing several brainwave frequencies, such as high alpha, low alpha, high beta, low beta, theta, delta, and gamma bands. Furthermore, signals linked to focus or meditation scores were also recorded. The EEG data were acquired at a sampling frequency of 512 Hz [49], whereas measured values were acquired on a per-second basis. A small microchip within the gadget processed and transmitted electrical impulses to the PC through Bluetooth technology. The EEG data displayed discernible patterns of brainwave activity characterized by various frequencies, such as high alpha, low alpha, high beta, low beta, theta, delta, and gamma. The aforementioned patterns were observed at regular one-minute intervals for each site. Subsequently, the overall averages were computed for the duration of the five-minute activity. The headset was equipped with an EEG e-Sense Metric, which enabled the classification of brainwave frequencies into focus or meditation categories. The statistics pertaining to meditation and focus ranged from 1 to 100 as determined by the EEG e-Sense Metric. These values were categorized as follows: 0–20 signified an extremely low level, 20–40 indicated a somewhat low level, 40–60 represented a moderate level, 60–80 suggested a somewhat high level, and 80–100 denoted a very high level [50]. The eSense Attention meter assessed participants' focus levels by quantifying the strength of their mental attention or focus. The eSense Meditation meter was employed to quantify the cognitive processes taking place in the brain and evaluate the level of mental relaxation or tranquility reported by each participant. Systolic blood pressure, diastolic blood pressure, and heart rate were assessed using an Omron HEM-7011 sphygmomanometer, which is a device manufactured by Omron in China. These measurements were taken both before and after the experiment. The Chinese versions of the STAI [51] and SDM [52] were used to assess psychological states. The SDM had a 13-point scale that included word pairs such as “comfortable–uncomfortable,” “relaxed–energized,” and “natural–artificial” sensations. The STAI was used to assess anxiety and consists of 20 items (e.g., “I feel relaxed,” for example) that participants rated on a four-point scale. The state anxiety score was computed by aggregating the responses to the 20 questions, with a higher score indicating elevated anxiety. Before and after each task, the subjects completed the STAI and SDM. For both time points, the overall process was the same.

### Statistical analysis

The statistical analysis was performed with SPSS 16.0 (SPSS Inc., Chicago, IL) software. Both analysis of variance (ANOVA) and a paired t test were used to assess the mean values of the physiological data. A *P* value of less than 0.05 was chosen as the cutoff point for statistical significance. The Wilcoxon signed-rank test was utilized to evaluate the psychological data, with a significance threshold of *P* < 0.01.

## Results

**Fig. 2:**
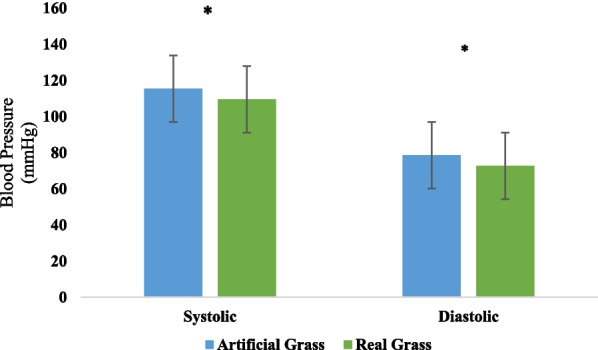
Comparison of systolic and diastolic blood pressure between real grass and artificial grass (control) tasks. *N* = 54; mean ± SE; *P < 0.05; by paired *t*-test

**Fig. 3:**
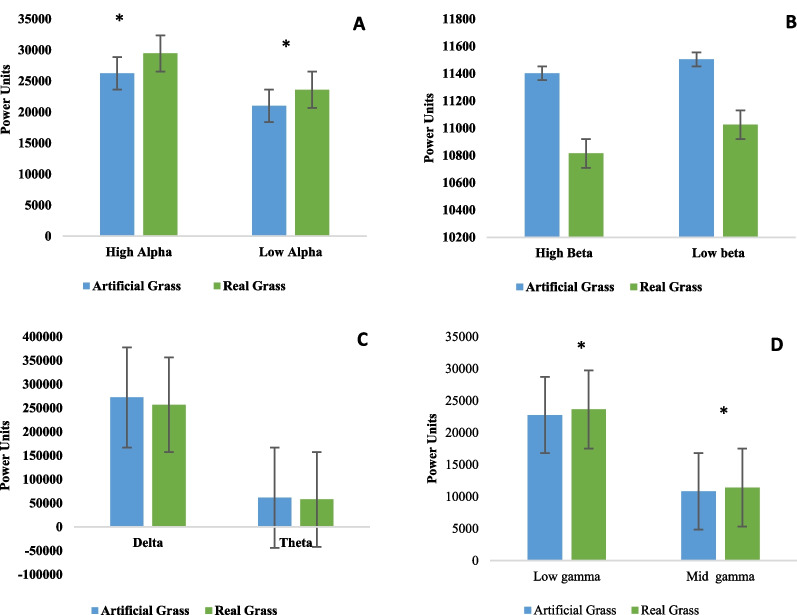
Comparison of overall (5 min) mean values of EEG-brainwaves with eye closed **A** Overall mean values for high and low alpha waves activity **B** Overall mean values for high and low beta waves activity **C** Overall mean values for delta and theta waves activity **D** Overall mean values for low gamma and mid gamma waves activity between real grass and artificial grass (control) tasks. *N* = 54, mean ± SE, paired *t* test, *P < 0.05

**Fig. 4:**
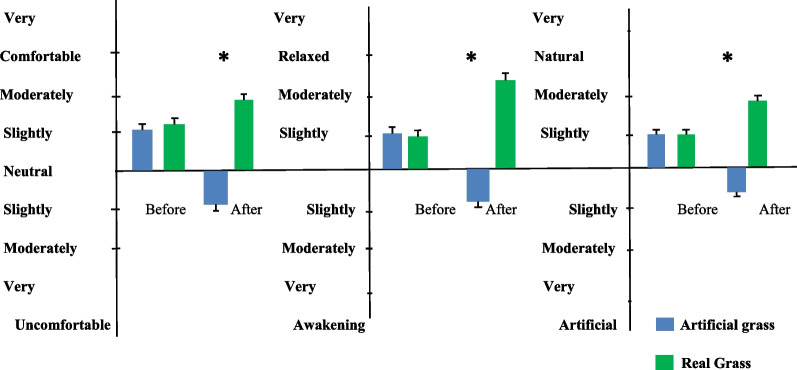
Participants scoring for comfortable, natural and relaxed feelings before and after touching real grass and artificial grass (control). *N* = 54, mean ± SE, *P < 0.01 by Wilcoxon signed-rank test

**Fig. 5:**
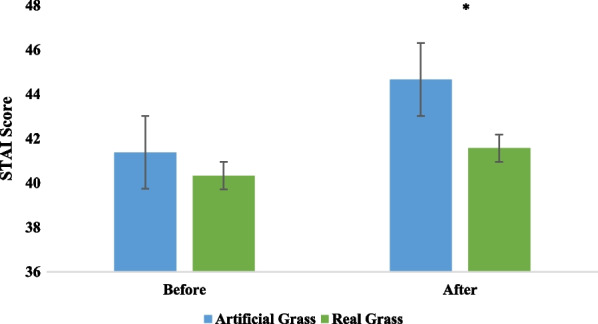
Comparison of participants’ STAI scores before and after real grass and artificial grass tasks (control). *N* = 54, mean ± SE, *P < 0.01 by Wilcoxon signed-rank test

The systolic (*P* = 0.002) and diastolic (*P* = 0.000) blood pressures of the participants exhibited a significant drop (*P* < 0.05) after 5 min of touching real grass (see Fig. 2). No significant (*P* > 0.05) difference in initial blood pressure values was observed between the two experimental conditions. There was not a significant (*P* > 0.05) variation in pulse rate (*P* = 0.97). The paired *t* test (Fig. 3) showed that tactile contact with real grass significantly (*P* < 0.05) increased activity in the high alpha (*P* = 0.0064), low alpha (*P* = 0.03), low gamma (*P* = 0.0078), and mid gamma (*P* = 0.03) bands compared to the control condition (touching artificial turf). However, there were no significant (*P* > 0.05) differences between the two conditions in terms of activity in the high beta (*P* = 0.21), low beta (*P* = 0.26), delta (*P* = 0.06), and theta (*P* = 0.11) bands, which indicates that these values were lower after 5 min of tactile contact with real grass than after 5 min of touching artificial turf. The findings from the one-way ANOVA indicated that touching real and artificial grass did not have a significant (*P* > 0.05) effect on activity in the high alpha (F = 0.4, DF = 9, *P* = 0.93), low alpha (F = 1.14, DF = 9, *P* = 0.33), high beta (F = 0.55, DF = 9, *P* = 0.83), and low beta (F = 1.03, DF = 9, *P* = 0.41) bands. Furthermore, there were no significant (*P* > 0.05) effects on activity in the theta (F = 1.55, DF = 9, *P* = 0.13) or delta (F = 1.72, DF = 9, *P* = 0.08) bands. There were no significant (*P* > 0.05) differences between conditions in terms of mid-gamma or low gamma activity (F = 0.86, DF = 9, *P* = 0.55 and F = 0.52, DF = 9, *P* = 0.85, respectively). The participants in the two groups demonstrated various degrees of relaxation and focus. The paired t test revealed that real grass and artificial turf conditions differed significantly (*P* < 0.05) in terms of relaxation (real grass: 56.07 ± 8.57; artificial turf: 48.10 ± 7.54) and focus scores (real grass: 67.01 ± 11.83; artificial turf: 59.64 ± 10.22). The SDM was used to evaluate the psychological responses of the participants regarding their feelings of naturalness, comfort, and relaxation during the two conditions. Figure 4 demonstrates that the SDM scores for "comfortable," "relaxed," and "natural" sensations were significantly (*P* < 0.01) different between the real and artificial grass conditions. The "comfortable" score indicated that participants felt more at ease after tactile contact with real grass than artificial turf (*P* < 0.01). In addition, participants felt significantly more relaxed after tactile contact with real grass than artificial turf (*P* < 0.01). After tactile contact with real grass, the participants felt a greater sense of naturalness than after touching artificial turf (*P* < 0.01). Finally, we observed a large difference in STAI scores between the two conditions. Compared to touching artificial turf, touching real grass led to lower anxiety levels (real grass: 41.57 ± 5.08 and control: 44.66 ± 5.11; *P* < 0.01, Fig. 5). Furthermore, there was no significant difference in STAI scores at baseline between the two conditions (*P* > 0.01).

## Discussion

The primary objective of the present investigation was to assess the potential stress-alleviating properties associated with touching real grass compared to artificial turf through tactile contact (palm-to-surface) for a duration of 5 min. The primary objective of this study was to assess the psychological and physiological reactions of Chinese adults. The results revealed a significant reduction in the average values of systolic and diastolic blood pressure after touching real grass compared to artificial turf. This suggests that the act of touching real grass led to greater relaxation. The results obtained in the present study are inconsistent with the findings of our previous research, which indicated that toe-to-grass contact for a duration of 10 min resulted in a greater reduction in both systolic and diastolic blood pressure than toe-to-wood contact [53]. However, these findings align with those of other studies that demonstrated a marked reduction in both systolic and diastolic blood pressure when individuals participated in horticulture activities compared to cognitive activities [54]. Lee et al. [30] reported comparable results in a prior investigation in which individuals engaged in plant-related tasks, specifically the transplantation of an indoor plant. The literature extensively documents the advantages of participating in physical activity for the management and prevention of hypertension [55]. Knowles et al. [56] found that individuals who engage in regular physical exercise demonstrate decreased systolic and diastolic blood pressures compared to those who engage in physical activity infrequently. Collectively, these data indicate that engaging in activities that entail physical contact with plants (such as grass) may reduce the likelihood of hypertension in young adults residing in urban environments. EEG presents a novel and contemporary scientific methodology for investigating stress, as it can reveal alterations in typical brainwave patterns when individuals are exposed to external stimuli [57]. In the current study, EEG data were collected. To examine the potential cognitive benefits of touching grass for reducing stress, participants were instructed to close their eyes and touch real grass or artificial turf (control condition) for five minutes. The findings revealed a significant increase in the high and low alpha activity after 5 min touching real grass. This suggests that individuals who touched real grass experienced a greater sense of relaxation compared to those who touched artificial turf [58]. These findings are in line with a prior experimental study that investigated the impact of horticultural activities with green plants and similarly showed an increase in alpha activity [32]. The participants in the present study exhibited reduced physiological arousal and greater relaxation and alertness while in contact with real grass. The results of our study align with previous research on examined the impact of green environments on sympathetic and EEG activity. Nakamura and Fujii [59] reported a large increase in the alpha and beta activity among participants who viewed an image depicting a natural hedge as opposed to those who viewed a picture of a concrete wall [60]. A study indicated that alpha waves are linked to a state of calm wakefulness [61]. The initial findings suggested that different sites exhibit frontal alpha asymmetry (FAA) during exposure to specific urban green spaces. These findings suggest that there is a higher likelihood of strong FAA in individuals in parks compared to a control site [62]. Prior studies have established a favorable association between alpha power and the subjective sensation of happiness [63]; conversely, a negative correlation has been observed between alpha power and the experience of despair. The potential correlation between enhanced alpha power and the manifestation of withdrawal symptoms, including but not limited to rage, restlessness, irritability, sleeplessness, tremor, aggression, and lack of appetite, warrants further investigation. In addition, alpha waves are prominent during periods of calmness and serenity [64]. Furthermore, an increased level of alpha wave activity has been linked to a condition characterized by profound relaxation [32]. On the other hand, the observed reductions in alpha activity within the control group suggest that individuals experienced heightened levels of stress while performing the task [54]. The outcomes of our study align with the conclusions of a prior investigation that observed that the act of touching wood led to a reduction in alpha activity [53]. Additionally, Thompson et al. [65] found that decreased alpha wave activity is linked to self-reported feelings of alertness, vigilance, tension, agitation, and visual attentiveness. The EEG data revealed different responses to different environmental stimuli, including variations in green and nongreen environments, as well as comparisons between historical and contemporary settings. The results revealed a significant correlation between the presence of vegetation and alpha power in the occipital region of the brain [66]. Nevertheless, there is a correlation between abnormally low alpha activity and transient ischemia episodes [32]. Additional evidence suggests a potential association between alpha activity and cognitive exertion. Greater cognitive exertion results in the inhibition of alpha oscillations, specifically originating from the frontal regions [67]. Thus, it may be inferred that 5 min of tactile contact with real grass resulted in heightened relaxation, as seen by elevated levels of both high and low alpha activity compared to those in the control group. Participants in the present study exhibited a reduction in both high and low beta activity after tactile contact with real grass, suggesting a decline in active focus and a drop in busy or anxious thoughts among the subjects. Beta activity has historically been associated with wakefulness, alertness, cognitive involvement, and conscious cognitive processing [68]. According to Miller et al. [69], the presence of beta waves characterized by low magnitudes and high frequencies is linked with instances of active, engaged, or apprehensive cognitive processes and focused attention. According to Grassini et al. [70], the observation of natural photographs led to an increase in alpha activity and a simultaneous decrease in beta activity. This pattern of results indicates that individuals allocated fewer attentional resources when viewing natural images as opposed to urban images. Beta brainwaves are typically dominant during various activities, including athletics, verbal communication, and focused listening [32]. High and low beta activity in participants was elevated after the completion of the control task, suggesting heightened states of alertness and cognitive engagement [71]. Prior studies have also demonstrated a correlation between high beta activity and heightened mental activity as well as between low beta activity and drowsiness [72]. Furthermore, our findings align with those of a prior investigation that explored the impacts of a blend of visual and olfactory stimuli derived from four distinct plant environments (namely, a lawn, rose garden, osmanthus garden, and pine forest) on physiological restoration with a specific focus on beta activity as well as overall perceived quality assessment. Nevertheless, it is crucial to acknowledge that the aforementioned amalgamation of stimuli did not have a substantial psychological impact. The findings of their study indicated that the utilization of multimodal stimuli resulted in more pronounced physiological improvements [73]. Hence, it may be inferred that the application of tactile stimuli, whether with real or artificial grass, has a significant impact on beta waves. The present study demonstrated that the tactile contact with real grass resulted in a reduction in delta activity, which may be attributed to the induction of drowsiness [74]. Delta rhythms are commonly observed during periods of deep, non-REM sleep, and their presence in awake recordings is typically indicative of tiredness. Nevertheless, tactile contact with synthetic turf was observed to result in heightened delta activity, which is associated with subconscious processes. Delta activity predominantly takes place during periods of deep sleep, also known as slow-wave sleep, in which dream experiences are absent [75]. The results of our study are inconsistent with previous studies and suggest that increased activity in the temporal lobes is associated with higher delta power and lower alpha power in other areas of the brain. This indicates a positive relationship between olfactory stimuli and improved sleep quality [76]. Perl et al. [77] discovered that the presentation of lavender stimuli leads to an increase in delta activity during nonrapid eye movement (NREM) sleep. The findings of their investigation demonstrated a favorable correlation between the duration of odor emission and the period of heightened delta activity, which aligns with our own research findings. In the current investigation, no significant variations in theta wave activity were found between tactile contact with real and artificial turf. The findings of this study indicate that, based on the established parameters, differences in the grass surface do not appear to have a significant impact on theta wave activity. It is postulated that theta waves may be indicative of initial stages of tiredness [78]. Theta activity is associated with the cognitive state commonly referred to as “daydreaming” as well as with enhanced creativity, mental imagery, and imagination [79]. According to Rowan and Tolunsky [80], diffuse theta activity is frequently observed in children. Theta waves are observed in individuals during periods of shallow sleep with closed eyelids or during states of profound relaxation, such as meditation or hypnosis [80]. Furthermore, this phenomenon is closely linked to profound internalization as well as various forms of introspective and contemplative activities that encompass physical, emotional, and critical thinking aspects [81]. The present study demonstrated that tactile contact with real grass resulted in a marked increase in low and mid gamma wave activity, and an elevation in gamma wave activity may be indicative of heightened levels of meditation [82]. The findings of the present investigation are consistent with earlier experiments demonstrating a correlation with general gardening activity and the transplantation of plants (specifically, yellow chrysanthemums). The gardening study demonstrated a marked increase in low and mid gamma wave activity during transplanting of plants as opposed to the transplanting activity conducted without plants [32]. In the field of neurophysiological research, Chien et al. [83] conducted an EEG study that compared the average response to laser-induced pain stimuli and nonpainful electrical somatosensory stimuli. The researchers observed fluctuations in delta/theta, alpha, beta, and gamma frequency ranges during pain and tactile experiments while controlling for attentional influences by employing randomized stimulus delivery [83]. This observation implies a potential correlation between elevated gamma activity and an enhanced state of meditation after touching real grass. The SDM and STAI scores revealed that participants had heightened feelings of "comfort" and "relaxation" after engaging in tactile contact with real grass compared to artificial turf. The findings of the present study provide empirical evidence of the psychological benefits of exposure to grass. The findings partially align with previous investigations by our research group. Our previous research indicated that participants who directly touched grass with the soles of their feet exhibited decreased levels of anxiety and reported better feelings in response, in contrast to those who touched wood [53]. The findings of this research indicate that touching grass with one's palm is a straightforward and readily available method for establishing a connection with the natural environment. Therefore, this practice has the potential to improve mental health and overall quality of life in contemporary society.

Nevertheless, it is important to acknowledge the numerous limitations of the current study. First, only young people from China were included. Future research should endeavor to examine individuals across a variety of ages, include a diverse array of landscape plants, trees, and flowers, and implement a more rigorously delineated control group. Second, the participants were not fully informed about the study's objectives to mitigate the potential for biased responses. While this approach may have helped obtain more natural reactions from the participants, it also posed ethical concerns and could impact the generalizability of the results. The lack of understanding about the study's purpose among participants might have led to varying levels of engagement or interest, potentially skewing the data or introducing uncontrolled variables. Thus, the findings should be interpreted cautiously and further studies may be needed in which the purpose is transparently communicated to participants.

## Conclusion

The findings of this research suggest that including tactile contact, such as with real grass, may promote relaxation among Chinese individuals by improving their psychophysiological state. The empirical evidence indicated greater relaxation in this condition according to decreases in systolic and diastolic blood pressure in addition to increased alpha and gamma activity. The findings of this study underscore the therapeutic advantages of touching natural grass and its potential to enhance general well-being and mitigate stress among Chinese adults who live and work in urban settings.

## Data Availability

Because it is confidential, the research data utilized to support the findings of this study have not been made public.
